# Enhanced effect of recombinant human soluble thrombomodulin by ultrasound irradiation in acute liver failure

**DOI:** 10.1038/s41598-020-58624-0

**Published:** 2020-02-03

**Authors:** Kota Hoshino, Yoshihiko Nakamura, Takafumi Nakano, Akiko Watanabe, Hong Sheng, Katsuro Tachibana, Hiroyasu Ishikura

**Affiliations:** 1 Department of Emergency and Critical Care Medicine, Faculty of Medicine, Fukuoka University, Fukuoka, Japan; 2 Department of Pharmacology, Faculty of Pharmaceutical Sciences, Fukuoka University, Fukuoka, Japan; 3 Department of Anatomy, Faculty of Medicine, Fukuoka University, Fukuoka, Japan

**Keywords:** Inflammation, Hepatology

## Abstract

The administration of recombinant human soluble thrombomodulin (rhsTM) significantly improves liver inflammation and increases the survival rate of patients with acute liver failure (ALF). However, rhsTM is dose-dependently correlated to the risk of bleeding. Recently, ultrasound (US) was found to enhance the effect of various drugs. Thus, the present study aimed to determine the enhancement effect of US irradiation on rhsTM in ALF. rhsTM (1 mg/kg) and US (1 MHz, 0.3 W/cm^2^) were irradiated to the liver of lipopolysaccharide/D-galactosamine-induced ALF mice model. The post-treatment aspartate aminotransferase, alanine aminotransferase, and high-mobility group box 1 levels were significantly lower in the rhsTM + US group than in the rhsTM alone group. Histopathological findings revealed significantly reduced liver injury and apoptosis in the rhsTM + US group. By contrast, US irradiation had no effect on rhsTM and TNF-α concentration in the liver tissue. In conclusion, US irradiation enhanced the effect of rhsTM in the ALF mice model. However, further studies must be conducted to determine the exact mechanism of such enhancement effect.

## Introduction

Thrombomodulin is a cell surface-expressed glycoprotein. This glycoprotein is a cofactor of protein C thrombin-mediated activation. Endothelial cell protein C receptor amplifies this pathway, thus attributing to a major anticoagulant mechanism that downregulates thrombin formation and thrombus inhibition^1^. Recombinant human soluble thrombomodulin (rhsTM) comprises the extracellular domain of thrombomodulin; thus, it has been used for the treatment of disseminated intravascular coagulation (DIC). A considerable amount of studies on the mechanisms underlying the therapeutic efficacy of rhsTM has been conducted. Moreover, the D1 domain of rhsTM bounds to the high-mobility group box 1 (HMGB1), which has potent anti-inflammatory effects via different molecular mechanisms^2,3^, thereby leading to the suppression of tumor necrosis factor (TNF-α) via the inhibition of macrophage activation^4^.

Acute liver failure (ALF) is initiated by the activation of inflammatory cells. It is known that macrophages release inflammatory cytokines which is a condition characterized by the rapid deterioration of hepatic cell function^5^. The overall survival of patients with ALF is 67%, and approximately 30% of patients with ALF undergo liver transplantation^6^. Osumi *et al*.^7^ have shown that the administration of rhsTM attenuated liver damage and increased survival rates in an ALF mice model. However, the dose of rhsTM (100 mg/kg, subcutaneous administration) in the previous study was significantly higher than that used in clinical settings (0.06 mg/kg). As the administration of rhsTM at a high dose may cause systemic bleeding complications in actual clinical practice, a method that reduces the dosage and enhances the effect of rhsTM on the target lesion is urgently needed.

Ultrasound (US) has been widely used as a clinical diagnostic tool. However, US has been developed not only as an imaging modality but also as a therapeutic tool in recent years^8–12^. High frequency US (1–10 MHz) and a range of intensities (0–20 W/cm^2^) focused on the tissue can increase the cell membrane permeability of therapeutic drugs. Thus, US can enhance the effect of drugs in a specific targeted area. US-targeted therapy has applications in gene therapy and anticancer drug delivery^13–15^. If US-targeted therapy can be utilised in ALF, such therapy can be considered a new method to decrease the dose of rhsTM to the liver, thereby preventing the risk of systemic bleeding complications.

The present study aimed to identify the enhancement effect of US irradiation on rhsTM in an ALF mice model. Moreover, the liver function, production of inflammatory mediators and rhsTM levels of the liver in the ALF model were evaluated to explore the mechanism on how rhsTM and US contribute to the regulation of liver inflammation.

## Methods

### Animal model

To induce ALF, male C57BL/6 (8 weeks) mice were injected with lipopolysaccharide (LPS; Escherichia coli, O111: B4) 4 µg/kg and D-galactosamine (GalN) 600 mg/kg intraperitoneally^16,17^. LPS and GalN were purchased from Sigma (St. Louis, MO). The mice were randomly assigned into six groups (n = 5): normal, placebo (LPS/GalN injected intraperitoneally and normal saline intravenously); rhsTM 1 mg/kg (LPS/GalN injected intraperitoneally and rhsTM intravenously); rhsTM 5 mg/kg; rhsTM 1 mg/kg + US; and rhsTM 5 mg/kg + US (Table 1). rhsTM was administered 30 min after LPS/GalN injection. rhsTM was obtained from Asahi Kasei Pharma Co. (Tokyo, Japan). US irradiation was carried out using the 10-mm diameter transducer (Sonitron 1000, Rich-Mar, USA) after the removal of hair with electrical clippers and application of Aquasonic 100 US gel on the abdominal skin. All US irradiations were performed separately at a frequency of 1 MHz and an intensity of 0.3 W/cm^2^ for 60 s (duty cycle, 50%) immediately after the administration of rhsTM. Blood samples were collected via cardiac venipuncture 7 h after LPS/GalN injection. Plasma samples were obtained via centrifugation of 1 ml of blood at 3000 rpm for 15 min and were frozen at −80 °C until use. The liver tissues of the sacrificed mice were obtained via laparotomy 7 h after LPS/GalN injection. The liver tissues were homogenised immediately after dissection from the left lobe of the liver. The time points of collecting plasma and liver tissue samples were based on a similar experiment reported by Osumi^7^. All experiments were approved by the Experimental Animal Care and Use Committee of Fukuoka University. All methods were performed in accordance with the Animal Care Guidelines of Fukuoka University.

**Table 1:** Detailed model description for each group.

Groups	LPS/GalN	rhsTM	US
Normal	—	—	—
Placebo	+	—	—
rhsTM 1 mg/kg	+	+	—
rhsTM 5 mg/kg	+	+	—
rhsTM 1 mg/kg + US	+	+	+
rhsTM 5 mg/kg + US	+	+	+

LPS: lipopolysaccharide, GalN: D-galactosamine, rhsTM: recombinant human soluble thrombomodulin, US: ultrasound.

### Evaluation of liver enzyme and HMGB1 levels in the plasma

The degree of liver dysfunction was evaluated via the measurement of plasma aspartate aminotransferase (AST) and alanine aminotransferase (ALT) levels (LSI Medience Co., Fukuoka, Japan). The plasma HMGB1 levels were measured using an enzyme-linked immunosorbent assay (ELISA; Shino-Test Co., Kanagawa, Japan) according to the manufacturer’s instructions. Briefly, the microplates were coated with a purified anti-HMGB1 antibody, which specifically binds to HMGB1. A diluent buffer (50 μL) was pipetted into the wells of the microtiter plate; subsequently, 50 μL of the standard, positive control and serum or plasma samples were added to each well. The plate was covered with an adhesive foil and incubated at 37 °C for 24 h to facilitate the binding of HMGB1 to the antibodies on the plate. Subsequently, the plate was washed five times and 100 μL of the enzyme conjugate was added. Once again, the plate was sealed and incubated for 2 h at room temperature. After washing, 100 μL of colour solution was added and incubated for 30 min at room temperature. Finally, 100 μL of stop solution was added, and the optical density was photometrically measured at 450 nm. The final concentration values were calculated using the optimised standard curve (eight-step dilution of 1:2 with an initial concentration of 20 ng/mL) included in the assay kit^18^.

### Histopathological assessment

The liver tissue from the left lobe was obtained at the time of sacrifice. The liver tissue was fixed in 10% buffered formalin (Muto Pure Chemicals Co., Ltd., Japan). The fixed liver tissues were embedded in paraffin and sectioned into 3-µm slices. The paraffin-embedded sections were stained with hematoxylin and eosin (HE) for pathological analysis (n = 3 in each group). All histological images were obtained with an optical microscope (BZ-X710, Keyence Corporation).

The HE-stained sections were evaluated for the severity of hepatic injury using the point-counting method with histological scores reported by Bak *et al*.^19^. Briefly, HE stained sections were graded as follows: grade 0, minimal or no evidence of injury; grade 1, mild injury consisting of cytoplasmic vacuolation and focal nuclear pyknosis; grade 2, moderate to severe injury with extensive nuclear pyknosis; and grade 3, severe necrosis with disintegration of hepatic cords, haemorrhage, and neutrophil infiltration.

Apoptosis was assessed using the terminal deoxynucleotidyl transferase-mediated dUTP nick-end labelling (TUNEL) assay Kit (Promega, Tokyo, Japan) according to the manufacturer’s instructions. The coverslips were mounted using Vectashield (Vector Laboratories), and the slides were observed under microscope. Then, three areas of the liver (upper, middle and lower part of the slide) (scale bar: 100 µm) were randomly selected and photographed under a microscope. The images were processed with NIH Image in a blinded manner for unbiased counting. The mean number of positively stained cell was calculated from three microscopic fields in each section of the liver, and the sections were analysed for each liver (n = 3 in each group). Data were expressed as the mean number of cells per square millimetre.

### TNF-α and rhsTM levels in the liver tissue

The TNF-α and rhsTM levels in the homogenised liver were measured using the TNF-α ELISA Kit (BioLegend, San Diego, USA) and Human Thrombomodulin/BDCA-3 Quantikine ELISA Kit (R&D Systems, Minnesota, USA) according to the manufacturer’s instructions.

### Statistical analysis

Data were presented as mean ± standard error of the mean (SEM) and were analysed using one-way analysis of variance, followed by Tukey’s post hoc test. A P value < 0.05 was considered statistically significant. All statistical analyses were conducted on a personal computer with the JMP software version 12 (SAS Institute, Cary, NC) for Windows.

## Results

### Evaluation of liver enzyme levels

**Figure 1:**
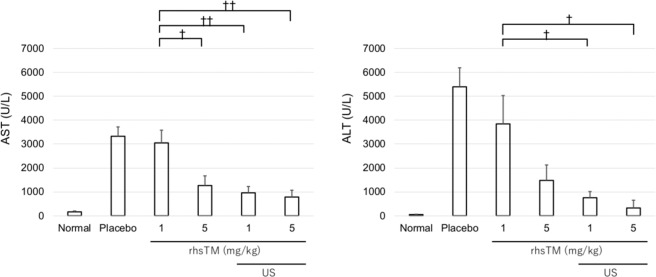
Plasma AST and ALT levels in the rhsTM and US irradiation groups 7 h after LPS/GalN injection (n = 5). Values were expressed as mean ± SEM. The Tukey’s test was performed without normal and placebo groups. ^†^P < 0.05, ^††^P < 0.01.

The AST and ALT levels of the normal, placebo, rhsTM 1 mg/kg, 5 mg/kg, 1 mg/kg + US and 5 mg/kg + US groups were 163 ± 37 and 48 ± 15 IU/L, 3324 ± 394 and 5391 ± 796 IU/L, 3047 ± 532 and 3841 ± 1187 IU/L, 1262 ± 408 and 1478 ± 645 IU/L, 955 ± 268 and 754 ± 258 IU/L and 783 ± 284 and 325 ± 324 IU/L, respectively (Fig. 1). Moreover, the AST and ALT levels of the US alone group (without the administration of rhsTM) were 4337 ± 749 and 4955 ± 1152 IU/L. The AST levels were significantly lower in the 5 mg/kg, 1 mg/kg + US, and 5 mg/kg + US rhsTM groups than in the 1 mg/kg rhsTM group (P < 0.05, P < 0.01 and P < 0.01). The ALT levels were significantly lower in the 1 mg/kg + US and 5 mg/kg + US rhsTM groups than in the 1 mg/kg rhsTM group (P < 0.05, respectively). The AST and ALT levels were lower in the 5 mg/kg + US rhsTM group than in the 5 mg/kg rhsTM group; however, no significant difference was observed between the 5 mg/kg + US and 5 mg/kg alone groups.

### HMGB1 level in the plasma

**Figure 2:**
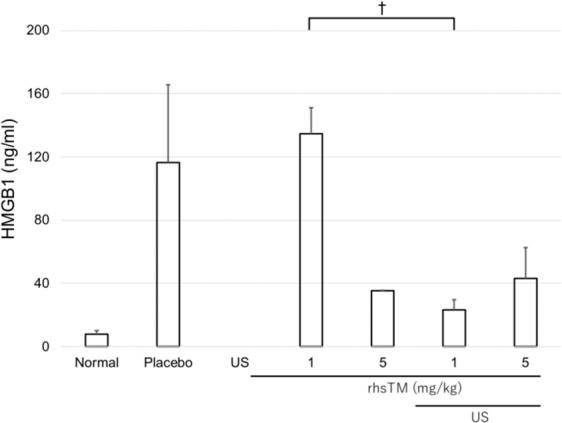
Plasma HMGB1 levels in the rhsTM and US irradiation groups 7 h after LPS/GalN injection (n = 5). Values were expressed as mean ± SEM. Tukey’s test was performed without normal and placebo groups. ^†^P < 0.05.

Figure 2 shows that the plasma HMGB1 level increased in the ALF model (placebo). The HMGB1 level in the 1 mg/kg rhsTM group did not sufficiently attenuate the administration of rhsTM. The HMGB1 level in the 1 mg/kg + US rhsTM group was significantly lower than that in the 1 mg/kg rhsTM group (23 ± 6 vs. 134 ± 16 ng/mL; P < 0.05). The HMGB1 levels were lower in the 5 mg/kg and 5 mg/kg + US rhsTM groups than in the 1 mg/kg rhsTM group; however, no significant differences were observed among all the groups.

### Histopathological assessment

**Figure 3:**
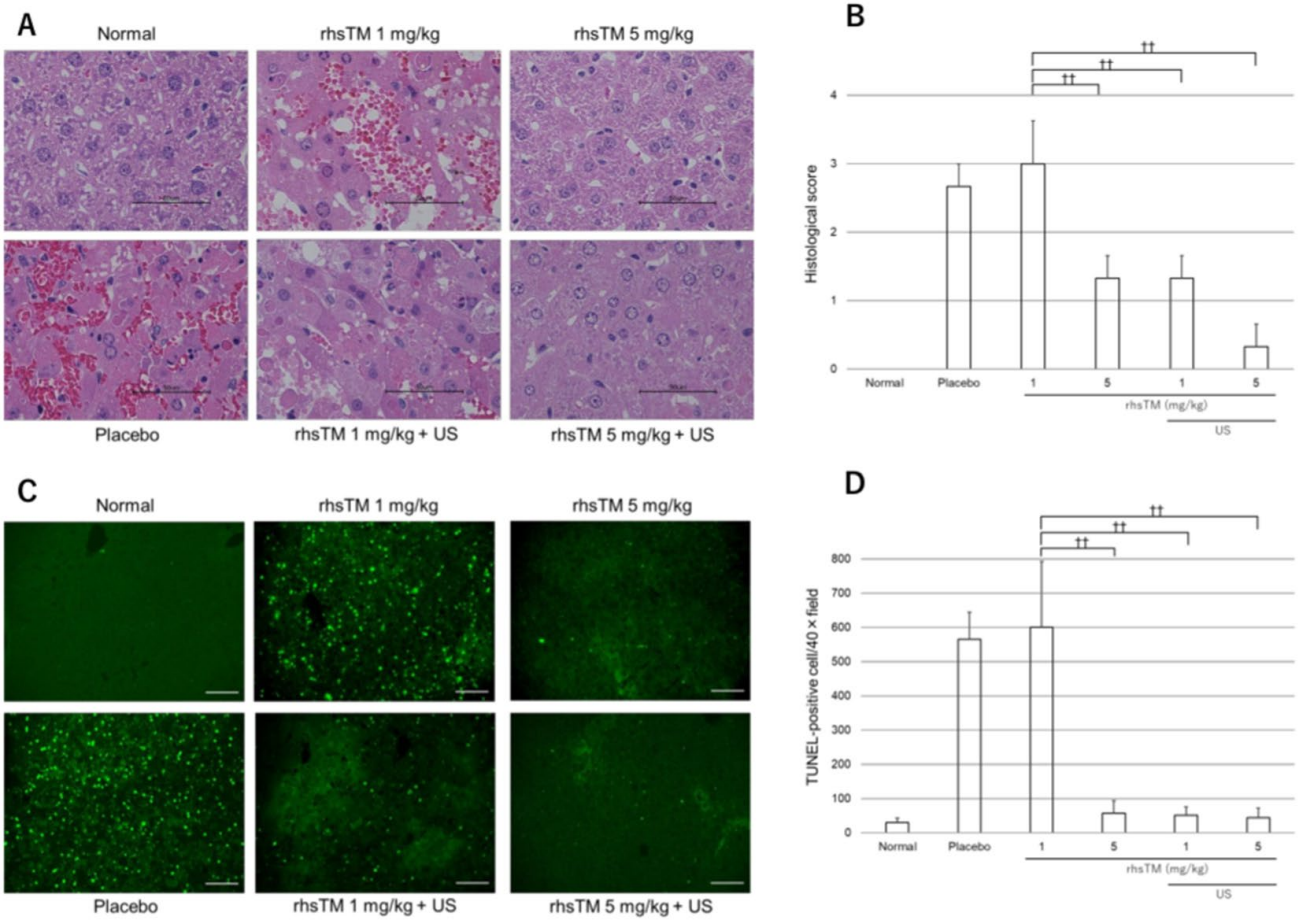
Panel A shows the representative HE-stained images (×60) in the left lobe of the liver in the rhsTM and US irradiation groups. Panel B indicates the histological score for evaluating the severity of hepatic injury (n = 3). Panel C shows the representative images of fluorescent double staining of terminal deoxynucleotidyl transferase-mediated dUTP nick-end labelling (TUNEL). Panel D shows the mean TUNEL-positive cell count in the three areas of the liver to evaluate for apoptosis (n = 3). Values were expressed as mean ± SEM. Tukey’s test was performed without normal and placebo groups. ^††^P < 0.01.

The histopathological findings of the liver in the rhsTM administered and US irradiated mice are shown in Fig. 3A (HE, ×60). In the placebo and 1 mg/kg groups, severe hepatic injury with extravasated red blood cells was observed. Moreover, lymphocytes were observed in the portal tracts and in the collapsed sinusoids. These lesions improved in the 5 mg/kg, 1 mg/kg + US, and 5 mg/kg + US groups. As shown in Fig. 3B, the histological score was calculated to evaluate the severity of hepatic injury in the ALF model. The histological score was significantly lower in the 5 mg/kg, 1 mg/kg + US, and 5 mg/kg + US rhsTM groups than in the 1 mg/kg rhsTM group (1.3 ± 0.3 vs. 3.0 ± 0.6; P < 0.01, 1.3 ± 0.3 vs. 3.0 ± 0.6; P < 0.01, and 0.3 ± 0.3 vs. 3.0 ± 0.6; P < 0.01). Moreover, we detected apoptosis cells in the liver tissues via TUNNEL staining to assess the effect of rhsTM administration in the ALF model (Fig. 3C). As shown in Fig. 3D, the TUNEL-positive cells significantly decreased in the 5 mg/kg, 1 mg/kg + US, and 5 mg/kg + US rhsTM groups than in the 1 mg/kg rhsTM group.

### TNF-α level in the liver tissue

**Figure 4:**
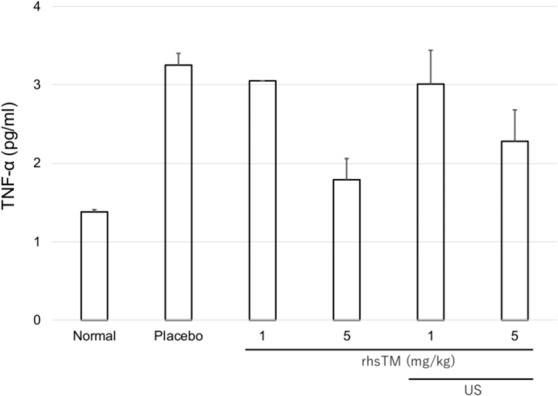
TNF-α levels in the liver of the rhsTM and US irradiation groups 7 h after LPS/GalN injection (n = 5). Values were expressed as mean ± SEM. Tukey’s test was performed without normal and placebo groups.

The TNF-α levels in the liver tissue increased in the placebo group (ALF model) compared to the normal group (Fig. 4). The TNF-α levels in the liver were lower in the 5 mg/kg group than in the 1 mg/kg group. However, no significant difference was observed. The TNF-α levels in the liver were not suppressed in the US irradiation groups.

### rhsTM levels in the liver tissue

**Figure 5:**
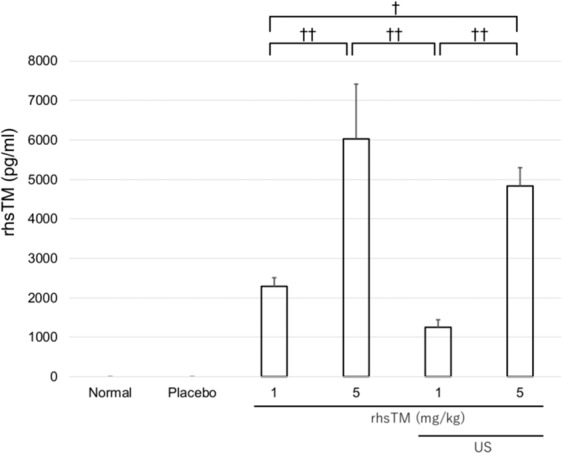
rhsTM levels in the liver of the rhsTM and US irradiation groups 7 h after LPS/GalN injection (n = 5). Values were expressed as mean ± SEM. Tukey’s test was performed without normal and placebo groups. ^†^P < 0.05, ^††^P < 0.01.

The presence of rhsTM in the liver tissue was not detected in the normal, placebo groups. The rhsTM levels in the liver were significantly higher in the 5 mg/kg (or + US) group than in the 1 mg/kg (or + US) group [6029 ± 1388 (4835 ± 465) vs. 2289 ± 218 (1250 ± 192) pg/mL; P < 0.01, respectively]. However, no change was observed in the rhsTM concentration in the liver tissue of the US irradiation group (Fig. 5).

## Discussion

Recent clinical studies have shown that the administration of rhsTM can reduce mortality by improving organ dysfunction^20,21^. In addition, the use of rhsTM was approved for the treatment of not only septic DIC but also for cancer, which is particularly important due to the expectation that the number of cancer cases will increase by more than 20% in 2020^22^. rhsTM has anticoagulant and anti-inflammatory effects. Therefore, such drug can be widely used for various diseases^7,23,24^. Moreover, it is available as a novel analgesic for the treatment of HMGB1-mediated inflammatory pain as peripheral HMGB1 plays important roles in the development of inflammatory hyperalgesia^25–27^.

The present study first evaluated the effects of rhsTM in an ALF model. According to this study, compared with an rhsTM dose of 1 mg/kg, a dose of 5 mg/kg significantly reduced the plasma AST levels and liver injury as well as apoptosis based on a histopathological assessment. This result could validate the findings of the previous study of Osumi *et al*.^7^ Nakamura *et al*.^24^ have also reported that rhsTM dose-dependently ameliorated cerebral ischaemia injury via the HMGB1 inhibitory mechanism in mice. Therefore, the administration of rhsTM at high dosages is more effective in treating severe liver inflammation.

rhsTM is currently administered at a dose of 0.06 mg/kg (380 U/kg) in clinical settings^28^. In case of renal failure, the administration of rhsTM is often decreased to 0.02 mg/kg (130 U/kg). The clinical doses of rhsTM are significantly lower than those reported in other animal models. Saito has shown that the incidence of bleeding-related adverse events in clinical settings was significantly lower in the rhsTM-treated group than in the heparin-treated group [50/116 patients (43.1%) vs. 65/115 patients (56.5%); P = 0.0487]^28^. This result indicated that rhsTM may have a wider safety margin than other anticoagulants. However, the percentage of bleeding-related adverse events was 43.1% in the rhsTM-treated group. In addition, in the rhsTM-treated group, the percentage of bleeding-related adverse events that lead to discontinuation of treatment was 1.7%^28^. rhsTM is associated with a risk of bleeding^29^. In addition, some studies have concluded that the risk of bleeding is dependent on the dose of rhsTM^30^. Thus, the administration of rhsTM at higher doses should be avoided due to the risk of bleeding. An alternative therapy is required to enhance the effect of rhsTM to a specific organ, particularly in the liver, without increasing the dosage.

Previously, irradiation via low-intensity US was found to generate small transient holes in the cell membrane and to increase the cell membrane permeability. This phenomenon is referred to as sonoporation. Some studies have shown that sonoporation increases the efficacy of anticancer drugs and gene delivery^31–33^. Sonoporation, which is a new method used for targeted drug delivery and non-viral gene transfection, has several advantages.

The present study aimed to evaluate the possible enhancement effects of US irradiation on rhsTM in the ALF model. The AST and ALT levels did not change in the US irradiation alone mice and had no effects on the liver, US irradiation after the administration of 1 mg/kg of rhsTM significantly decreased the plasma AST, ALT and HMGB1 levels. By contrast, rhsTM at a dose of 5 mg/kg in combination with US decreased the plasma AST and ALT levels compared to 5 mg/kg alone in the treated mice. Although the results were not statistically significant, US irradiation after the administration of rhsTM was not considered completely ineffective. As the plasma AST and ALT levels of the rhsTM 5 mg/kg group decreased sufficiently, the statistical power was not enough between the 5 mg/kg and 5 mg/kg + US groups.

Our results showed that the histological score and number of TUNEL-positive cells were significantly lower in the 1 mg/kg + US group than in the 1 mg/kg group. To support the enhancement effect of rhsTM via US irradiation, US irradiation after the administration of rhsTM revealed improvement of liver injury and apoptosis according to the histopathological assessment. Therefore, these observations indicated that US irradiation enhanced the effect of rhsTM.

A significant increase of TNF-α level in the liver has been previously observed 1 h after LPS/GalN injection^34,35^. Furthermore, TNF-α levels in the liver showed an early peak at 1 h and then decreased^7^. In this study, the liver samples were obtained 7 h after LPS/GalN injection according to the previous study^7^. The liver TNF-α levels were lower in the 5 mg/kg group than in the 1 mg/kg group. However, no significant difference was observed. This result has inadequate statistical power to detect significant differences in the dose-dependently effect between the 1 mg/kg and 5 mg/kg groups. Moreover, we evaluated the TNF-α levels in the liver to elucidate the enhancement effect of rhsTM via US irradiation. No significant differences in the TNF-α level in the liver were observed between the 1 mg/kg and 1 mg/kg + US groups as well as in the 5 mg/kg and 5 mg/kg + US groups. In other words, the enhancement effect of US irradiation was not detected based on the TNF-α levels in the liver tissue. Although increased levels of rhsTM in the liver via US irradiation were expected due to the possible mechanism of sonoporation, US irradiation may not have affected the increase in rhsTM concentration in the liver. Thus, US irradiation enhanced the effect of rhsTM in the plasma based on the histopathological assessment; however, the mechanism of sonoporation could not be clearly proven in our study.

The cell membrane consists of a lipid bilayer with embedded proteins. Phosphatidylserine (PS) is a phospholipid and is a component of the cell membrane. PS is normally confined to the inner leaflet; however, it is transferred to the outer leaflet via the activity of the scramblase enzymes^36^. This mechanism is known as PS externalisation, which facilitates coagulation. Moreover, PS externalisation is involved in the recognition of apoptotic cells^37,38^. Ruijssevelt has reported that the mechanism of the enhancement effect of US irradiation was not correlated to sonoporation and was dependent on regulated PS externalisation *in vitro*^39^. As our results revealed that rhsTM levels in the liver did not increase *in vivo*, it is consistent with the previous study of regulated PS externalisation. However, this hypothesis cannot be completely proven in this limited evaluation. Multiple drug concentration measurements should be conducted at various time points to fully understand the pharmakinetics within the liver tissue after US irradiation.

The limitation of the present study can be summarized as follows. Firstly, pro-inflammatory cytokines should have been measured to assess the mechanism of rhsTM and US irradiation in more detail. Secondly, the optimal timing of rhsTM administration is unknown. It is estimated from a previous study that the level of pro-inflammatory cytokines started to increase 1 h after LPS/GalN injection^7^. Therefore, we administered rhsTM 30 min after LPS/GalN injection in our experiments. Thirdly, although rhsTM doses of 1 mg/kg or 5 mg/kg used in our experiment is based on previously reported rhsTM studies, it is uncertain if these dosages have clinical relevance. Anti-inflammatory effects of rhsTM at these dosages had a significant effect on the prevention of lung injury in a rat model with LPS-induced systemic inflammation^40^. In a mouse heat stroke model, significant amelioration of liver injury was observed with the administration of 1 mg/kg of rhsTM^41^. The same dosages were applied to ameliorated cerebral ischaemic injury model without haemorrhagic complications in mice^24^. However, all the above doses of rhsTM were significantly higher than that used in clinical settings (0.06 mg/kg). Nevertheless, further studies should be performed to evaluate on these above limitations if our method were to be used for patients.

## Conclusions

After the administration of rhsTM, low-intensity US irradiation reduced liver enzyme levels, HMGB1 level as well as liver injury and apoptosis in the ALF model. This result indicated that US irradiation enhances the effect of rhsTM.

## References

[1] Anastasiou G, Gialeraki A, Merkouri E, Politou M, Travlou A (2012). Thrombomodulin as a regulator of the anticoagulant pathway: implication in the development of thrombosis. Blood Coagul Fibrinolysis..

[2] Abeyama K (2005). The N-terminal domain of thrombomodulin sequesters high-mobility group-B1 protein, a novel antiinflammatory mechanism. J. Clin. Invest..

[3] Ito Takashi, Kawahara Ko-ichi, Okamoto Kohji, Yamada Shingo, Yasuda Minetsugu, Imaizumi Hitoshi, Nawa Yuko, Meng Xiaojie, Shrestha Binita, Hashiguchi Teruto, Maruyama Ikuro (2008). Proteolytic Cleavage of High Mobility Group Box 1 Protein by Thrombin-Thrombomodulin Complexes. Arteriosclerosis, Thrombosis, and Vascular Biology.

[4] Grey ST (1994). Selective inhibitory effects of the anticoagulant activated protein C on the responses of human mononuclear phagocytes to LPS, IFN-gamma, or phorbol ester. J Immunol..

[5] Schwabe RF, Seki E, Brenner DA (2006). Toll-like receptor signaling in the liver. Gastroenterology..

[6] Ostapowicz G (2002). Results of a prospective study of acute liver failure at 17 tertiary care centers in the United States. Ann Intern Med..

[7] Osumi W (2015). Recombinant human soluble thrombomodulin improved lipopolysaccharide/d-galactosamine-induced acute liver failure in mice. J. Pharmacol. Sci..

[8] Wang T-Y, Wilson K, Machtaler S, Willmann J (2014). Ultrasound and Microbubble Guided Drug Delivery: Mechanistic Understanding and Clinical Implications. Curr. Pharm. Biotechnol..

[9] Mullick Chowdhury S (2016). Ultrasound-guided therapeutic modulation of hepatocellular carcinoma using complementary microRNAs. J Control Release..

[10] Taniyama Y (2002). Local delivery of plasmid DNA into rat carotid artery using ultrasound. Circulation.

[11] Taniyama Y (2002). Development of safe and efficient novel nonviral gene transfer using ultrasound: Enhancement of transfection efficiency of naked plasmid DNA in skeletal muscle. Gene Ther..

[12] Tachibana K, Uchida T, Ogawa K, Yamashita N, Tamura K (1999). Induction of cell-membrane porosity by ultrasound. Lancet..

[13] Narihira K (2018). Enhanced cell killing and apoptosis of oral squamous cell carcinoma cells with ultrasound in combination with cetuximab coated albumin microbubbles. J Drug Target..

[14] Chang S (2013). Targeted microbubbles for ultrasound mediated gene transfection and apoptosis induction in ovarian cancer cells. Ultrason. Sonochem..

[15] Escoffre JM, Piron J, Novell A, Bouakaz A (2011). Doxorubicin delivery into tumor cells with ultrasound and microbubbles. Mol Pharm..

[16] Galanos C, Freudenberg MA, Reutter W (1979). Galactosamine-induced sensitization to the lethal effects of endotoxin. Proc. Natl. Acad. Sci. USA.

[17] Imai Y (2014). Chymase inhibition attenuates lipopolysaccharide/ d-galactosamine-induced acute liver failure in hamsters. Pharmacology..

[18] Lehner J (2012). Methodological and preanalytical evaluation of an HMGB1 immunoassay. Anticancer Res..

[19] Bak DH (2018). Anti-apoptotic effects of human placental hydrolysate against hepatocyte toxicity *in vivo* and *in vitro*. Int J Mol Med..

[20] Hayakawa M (2016). Recombinant human soluble thrombomodulin and mortality in sepsis-induced disseminated intravascular coagulation. A multicentre retrospective study. Thromb Haemost..

[21] Yamakawa K (2015). Recombinant human soluble thrombomodulin in severe sepsis: A systematic review and meta-analysis. J. Thromb. Haemost..

[22] Weir HK, Thompson TD, Soman A, Møller B, Leadbetter S (2015). The past, present, and future of cancer incidence in the United States: 1975 through 2020. Cancer..

[23] Irie Y (2017). Macrophage-derived HMGB1 as a Pain Mediator in the Early Stage of Acute Pancreatitis in Mice: Targeting RAGE and CXCL12/CXCR4 Axis. J Neuroimmune Pharmacol..

[24] Nakamura Y (2016). Recombinant human soluble thrombomodulin ameliorates cerebral ischemic injury through a high-mobility group box 1 inhibitory mechanism without hemorrhagic complications in mice. J Neurol Sci..

[25] Nishida Takeshi, Tsubota Maho, Kawaishi Yudai, Yamanishi Hiroki, Kamitani Natsuki, Sekiguchi Fumiko, Ishikura Hiroyasu, Liu Keyue, Nishibori Masahiro, Kawabata Atsufumi (2016). Involvement of high mobility group box 1 in the development and maintenance of chemotherapy-induced peripheral neuropathy in rats. Toxicology.

[26] Tanaka Junichi, Yamaguchi Kaoru, Ishikura Hiroyasu, Tsubota Maho, Sekiguchi Fumiko, Seki Yukari, Tsujiuchi Toshifumi, Murai Akira, Umemura Takehiro, Kawabata Atsufumi (2014). Bladder pain relief by HMGB1 neutralization and soluble thrombomodulin in mice with cyclophosphamide-induced cystitis. Neuropharmacology.

[27] Tanaka J (2013). Recombinant human soluble thrombomodulin prevents peripheral HMGB1-dependent hyperalgesia in rats. Br. J. Pharmacol..

[28] SAITO H., MARUYAMA I., SHIMAZAKI S., YAMAMOTO Y., AIKAWA N., OHNO R., HIRAYAMA A., MATSUDA T., ASAKURA H., NAKASHIMA M., AOKI N. (2006). Efficacy and safety of recombinant human soluble thrombomodulin (ART-123) in disseminated intravascular coagulation: results of a phase III, randomized, double-blind clinical trial. Journal of Thrombosis and Haemostasis.

[29] Mimuro J (2013). Impact of recombinant soluble thrombomodulin (thrombomodulin alfa) on disseminated intravascular coagulation. Thromb Res..

[30] Tanaka Kosuke, Tawara Shunsuke, Tsuruta Kazuhisa, Hoppensteadt Debra, Fareed Jawed (2018). Pharmacological Differentiation of Thrombomodulin Alfa and Activated Protein C on Coagulation and Fibrinolysis In Vitro. Clinical and Applied Thrombosis/Hemostasis.

[31] Miller D, Bao S, Morris J (1999). Sonoporation of cultured cells in the rotating tube exposure system. Ultrasound Med. Biol..

[32] Ross JP, Cai X, Chiu JF, Yang J, Wu J (2002). Optical and atomic force microscopic studies on sonoporation. J Acoust Soc Am..

[33] Ward M, Wu J, Chiu J (1999). Ultrasound-induced cell lysis and sonoporation enhanced by contrast agents. J. Acous. Soc. Am..

[34] Imai Y (2014). Chymase inhibition attenuates lipopolysaccharide/d-galactosamine-induced acute liver failure in hamsters. Pharmacology..

[35] Kim SJ, Kim JK, Lee DU, Kwak JH, Lee SM (2010). Genipin protects lipopolysaccharideinduced apoptotic liver damage in D-galactosamine-sensitized mice. Eur J Pharmacol..

[36] Hankins HM, Baldridge RD, Xu P, Graham TR (2015). Role of Flippases, Scramblases and Transfer Proteins in Phosphatidylserine Subcellular Distribution. Traffic.

[37] Blankenberg FG (2008). *In vivo* imaging of apoptosis. Cancer Biology & Therapy..

[38] Blankenberg FG (2009). Imaging the molecular signatures of apoptosis and injury with radiolabeled annexin V. Proc. Am. Thorac. Soc..

[39] Van Ruijssevelt L (2013). Observations on the viability of C6-glioma cells after sonoporation with low-intensity ultrasound and microbubbles. IEEE Trans Ultrason Ferroelectr Freq Control..

[40] Hagiwara S (2010). *In vivo* and *in vitro* effects of the anticoagulant, thrombomodulin, on the inflammatory response in rodent models. Shock..

[41] Kawasaki T, Okamoto K, Kawasaki C, Sata T (2014). Thrombomodulin improved liver injury, coagulopathy, and mortality in an experimental heatstroke model in mice. Anesth Analg..

